# Alcohol

**Published:** 1903-04

**Authors:** 


### Correspondence.

### Alcohol.

*Editor Texas Medical Journal.*

DEAR SIR: Three times during the last half century medical manifestoes have been issued giving the opinion of physicians on alcohol. The first was issued in 1839, and was signed by eighty-six persons. The second was in 1847, and was signed by 2000 physicians, and the third appeared in 1871, with the signatures of over 4000 physicians, including the names of many leading physicians in all parts of the world. A fourth declaration of opinions is now being circulated for signatures, and reads as follows:

"The following statement has been agreed upon by the Council of the British Medical Temperance Association, The American Medical Temperance Association, the Society of Medical Abstainers in Germany, and leading physicians in England and on the continent. The purpose of this is to have a general agreement of opinions of all prominent physicians in civilized countries concerning the dangers from alcohol, and in this way give support to the efforts made to check and prevent the evils from this source.

"In view of the terrible evils which have resulted from the consumption of alcohol, evils which, in many parts of the world, are rapidly increasing, we, members of the medical profession, feel it to be our duty, as being in some sense the guardians of the public health, to speak plainly of the nature of alcohol, and of the injury to the individual, and the danger to the community which arise from the prevalent use of intoxicating liquors as beverages.

"We think it ought to be known that:

"1. Experiments have demonstrated that, even a small quantity of alcoholic liquor, either immediately or after a short time, prevents perfect mental action, and interferes with the function of the cells and tissues of the body, impairing self-control by producing progressive paralysis of the judgment and of the will, and having other markedly injurious effects. Hence, alcohol must be regarded as a poison, and ought not to be classed among foods.

"2. Observation establishes the fact that a moderate use of alcoholic liquors, continued over a number of years, produces a gradual deterioration of the tissues of the body, and hastens the change which old age brings, thus increasing the average liability to disease (especially to infectious diseases) and shortening the duration of life.

"3. Total abstainers, other conditions being similar, can perform more work, possess greater powers of endurance, have on the average, less sickness, and recover more quickly than non-abstainers, especially from infectious diseases, while they altogether escape diseases specially caused by alcohol.

"4. All the bodily functions of a man, as of every other animal, are best performed in the absence of alcohol, and any supposed experience to the contrary is founded on delusion, a result of the action of alcohol on the nerve centers.

"5. Further, alcohol tends to produce in the offspring of drinkers an unstable nervous system, lowering them mentally, morally, and physically. Thus, deterioration of the race threatens us, and this is likely to be greatly accelerated by the alarming increase of drinking among women, who have hitherto been little addicted to this vice. Since the mothers of the coming generation are thus involved, the importance and danger of this increase cannot be exaggerated.

"Seeing, then, that the common use of alcoholic beverages is always and everywhere followed, sooner or later, by moral, physical and social results of a most serious and threatening character, and that it is the cause, direct or indirect, of a very large proportion of the poverty, suffering, vice, crime, lunacy, disease, and death, not only in the case of those who take such beverages, but in the case of others who are unavoidably associated with them, we feel warranted, nay, compelled to urge the general adoption of total abstinence from all intoxicating liquors as beverages, as the surest, simplest and quickest method of removing the evils which necessarily result from their use. Such a course is not only universally safe, but is also natural.

"We believe that such an era of health, happiness, and prosperity would be inaugurated thereby that many of the social problems of the present age would be solved."

This declaration has already received the signatures of over 1000 physicians in all parts of the country. I have been appointed chairman to present this manifesto to American physicians for their endorsement. I should be very glad to receive the name, title and address of any physician who is willing to aid by his signature, to correct public sentiment and assist in the prevention of one of the great evils of the age. This is purely a scientific effort for the purpose of having a general consensus of opinion of the leading physicians of the world, and it is assumed that American physicians are equally enthusiastic and prompt to lend their signatures to this statement, as in the wine-drinking countries of Europe. A postal card, with address and title is earnestly solicited from every medical man who would like to be represented in this great movement for a clearer comprehension of the subject.

Address,

T. D. Crothers,
Hartford, Conn.

---

"The Cairo."
Washington, D. C., February 19, 1903.

*Editor of Texas Medical Journal.*

Dear Sir: The enclosed bill, with report, has been unanimously reported by the House Judiciary Committee, and by the Committee on Judiciary of the Senate. This, or a similar bill, has been introduced by Senators Hoar, Nelson, Bacon, McComas, Penrose, Quay, Money and Pettigrew, and Representatives Ray of New York, and Henry of Connecticut. Every effort is being made by Senators Hoar, and Simon of Oregon, and Representatives DeArmond, Jenkins and Powers of Massachusetts, to have the bill passed this session. All outside help from high sources is especially needed at this time. If the bill meets with your approval, will you kindly notice it editorially as soon as possible, and if not too much trouble, send me a copy of the issue.

Thanking you for anything you can do, I am

Very truly yours,

A. MacDonald,
Per D.

